# Key players in syntrophic propionate oxidation revealed by metagenome-assembled genomes from anaerobic digesters bioaugmented with propionic acid enriched microbial consortia

**DOI:** 10.3389/fmicb.2022.968416

**Published:** 2022-11-17

**Authors:** Minjae Kim, Chaeyoung Rhee, Michael Wells, Juhee Shin, Joonyeob Lee, Seung Gu Shin

**Affiliations:** ^1^Natural Resource Ecology Laboratory, Colorado State University, Fort Collins, CO, United States; ^2^Department of Energy Engineering, Future Convergence Technology Research Institute, Gyeongsang National University, Jinju, South Korea; ^3^Division of Earth Environmental System Science, Department of Environmental Engineering, Pukyong National University, Busan, South Korea; ^4^Department of Energy System Engineering, Gyeongsang National University, Jinju, South Korea

**Keywords:** anaerobic digestion, bioaugmentation, metagenome-assembled genome, propionate, syntrophism, novel species

## Abstract

Propionic acid (HPr) is frequently accumulated in anaerobic digesters due to its thermodynamically unfavorable degradation reaction. Here, we identify key players in HPr oxidation and organic overloading recovery from metagenome-assembled genomes (MAGs) recovered from anaerobic digesters inoculated with HPr-enriched microbial consortia before initiating organic overloading. Two independent HPr-enrichment cultures commonly selected two uncultured microorganisms represented with high relative abundance: *Methanoculleus* sp002497965 and JABUEY01 sp013314815 (a member of the *Syntrophobacteraceae* family). The relative abundance of JABUEY01 sp013314815 was 60 times higher in bioaugmented bioreactors compared to their unaugmented counterparts after recovery from organic overloading. Genomic analysis of JABUEY01 sp013314815 revealed its metabolic potential for syntrophic propionate degradation when partnered with hydrogenotrophic methanogens (e.g., *Methanoculleus* sp002497965) *via* the methylmalonyl-CoA pathway. Our results identified at least two key species that are responsible for efficient propionate removal and demonstrate their potential applications as microbial cocktails for stable AD operation.

## Introduction

Anaerobic digestion (AD) is widely used to treat various organic pollutants from wastewater because of its ability to mineralize organic matter whilst simultaneously generating energy for diverse microbial communities. Several different groups of prokaryotes carry out four metabolic steps in AD. Briefly, hydrolytic and acidogenic bacteria break down complex organic molecules into volatile fatty acids (VFAs, e.g., butyric acid, propionic acid, acetic acids, etc.), dihydrogen (H_2_), and carbon dioxide (CO_2_) *via* hydrolysis, acidogenesis, and acetogenesis (Hardy et al., 2021). Then, acetoclastic (e.g., *Methanosaetaceae*, *Methanosarcinaceae*) and hydrogenotrophic (e.g., *Methoanomicrobiales*, *Methanobacteriales*) methanogenic archaea produce CH_4_ from acetate and H_2_/CO_2_, respectively. Among VFAs, propionic acid (HPr) is especially recalcitrant to AD because the oxidation of HPr to H_2_ is thermodynamically unfavorable under standard conditions (ΔG^0^ = +76.1 kJ; Table 1; Dyksma and Gallert, 2019). Thus, this reaction depends on the syntrophic interaction of hydrogenotrophic methanogens, who can maintain a low H_2_ partial pressure sufficient to drive HPr oxidation, and Hpr oxidizing bacteria (Dyksma and Gallert, 2019).

**Table 1:** Reaction table.

Reaction	Oxidation equation	ΔG^0^ (KJ/rxn)
Acetate oxidation	C_2_H_3_O_2(aq)_ + H_2_O → HCO_3_^−^ + CH_4_	−31.0
Butyrate oxidation	C_4_H_7_O_2(aq)_ + 2H_2_O → 2C_2_H_3_O_2(aq)_ + H^+^ + 2H_2_	+48.1
Propionate oxidation	C_3_H_5_O_2(aq)_ + 2H_2_O → C_2_H_3_O_3(aq)_ + CO_2(g)_ + 3H_2(g)_	+76.1
Hydrogenotrophic methanogenesis	4H_2(g)_ + CO_2(g)_ → CH_4(g)_ + 2H_2_O_(l)_	−125.9
Syntrophic oxidation of propionate	C_3_H_5_O_2(aq)_ + 1/2H_2_O_(l)_ → C_2_H_3_O_3(aq)_ + 3/4CH_4(g)_ + 1/4CO_2(g)_	−26.0

To date, two different HPr oxidation pathways have been reported: the methylmalonyl-CoA pathway for HPr oxidation to acetate, H_2_ and CO_2_ (Plugge et al., 1993) and the C6 dismutation pathway to acetic acid and butyric acid, where butyric acid is eventually oxidized to H_2_ and acetic acid by other microbial community members (de Bok et al., 2001). A few species of the *Desulfobacterota* phylum (e.g., *Syntrophobacter* and *Pelotamaculum*) and *Desulfotomaculum* have been reported to use the methylmalonyl-CoA pathway, whereas only the bacterium *Smithella propionica*, also from the *Desulfobacterota* phylum, is known to use the C6 dismutation pathway (Dyksma and Gallert, 2019). Additionally, uncultured *Cloacimonetes* closely related to *Candidatus C. acidaminovorans* express methylmalonyl-CoA pathway genes with high homology to those of *Pelotomaculum* in a methanogenic bioreactor system (Nobu et al., 2015). This demonstrates that a previously unappreciated diversity of uncultured bacteria can mediate syntrophic HPr oxidation in natural anoxic environments and engineered ecosystems (e.g., AD).

HPr oxidation has been considered a rate-limiting step, and its accumulation is an indicator of unstable and even fatal AD processes during wastewater treatment (Amani et al., 2011). However, HPr is a chief intermediate in the flow of organic carbon to CH_4_, accounting for up to 35% of the total carbon source driving methanogenesis (Glissmann and Conrad, 2000). Therefore, developing efficient mechanisms for HPr oxidation is essential for the stable and reliable operation of AD processes. In our previous study, we demonstrated that a ‘one-shot’ bioaugmentation of ADs with HPr-enriched microbial communities showed the fastest recovery responses to organic overloading (i.e., ~25% recovery time and > 10% CH_4_ conversion efficiency compared to the control) among several different biological and non-biological supplementation strategies (Rhee et al., 2021b). However, the key microorganisms driving HPr oxidation and the potential functions that are responsible for the superior recovery responses of the bioaugmented anaerobic digesters we observed remain unknown.

In this study, we expanded upon and enriched our previous work by applying deep metagenome sequencing, resulting in the recovery of 200 high-quality metagenome-assembled genomes (MAGs; i.e., Quality = Completeness – 5* contamination >50) representing >83% of the total microbial communities in our anaerobic digesters. Our analysis of these MAGs reveals novel microbes and their metabolic potential for pathways directly responsible for the efficient removal of HPr and concomitant methane production after the anaerobic digesters received an organic shock.

## Materials and methods

### Bioreactor operation and two enrichments

**Figure 1:**
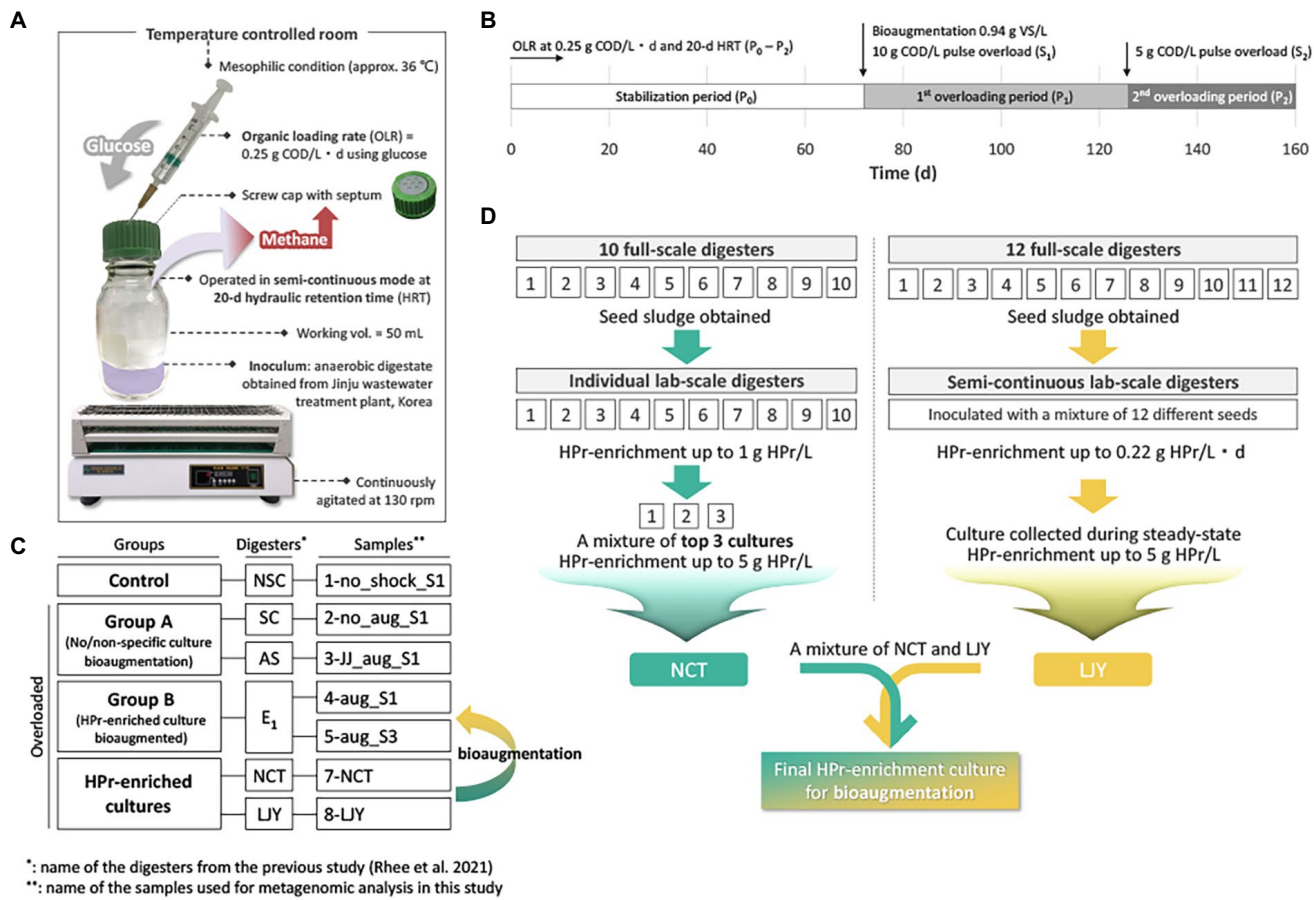
**(A)** The exprimental setup for the semi-continuous AD bioreactor tests. **(B)** Overview of the experimental timeline. Detailed methods for the AD operation can be found in (Rhee et al., 2021b). OLR, organic loading rate; COD, chemical oxygen demand; VS, volatile solids; HRT, hydraulic retention time. **(C)** Overview of the sample information. Please see the methods and Table 2 for the details. **(D)** Strategy of two HPr-enrichment cultures preparation and detailed information can be found in (Kim et al., 2015; Jannat et al., 2021; Lee et al., 2021).

In our previous study, the effect of bioaugmentation using HPr-enriched microbial consortia was investigated in overloaded semi-continuous anaerobic digesters as shown in Figure 1A (Rhee et al., 2021b). All digesters were triplicated and overloaded with two serial pulse feedings at 72 days (10 g COD/L; S_1_) and 126 days (5 g COD/L; S_2_) using glucose (Figure 1B), except for NSC digester which received no overloading as a control group (Figure 1C). Each of the overloaded digesters was treated with their assigned recovery strategy at 72 days, i.e., SC, no treatment after overload; AS, bioaugmentation with anaerobic sludge; E_1_, bioaugmentation with a mixture of NCT and LJY HPr-enriched cultures (Figure 1C). The two bioaugmented digesters AS and E_1_ were supplemented with non-specific conventional anaerobic sludge or HPr-enriched consortia as a pulse (0.94 g VS/L) at S_1_, respectively. Total seven samples for DNA extraction were taken from each digester at either 40 days after S1 (designated as ‘_S1’) or 30d after S_2_ (‘_S3’), which were considered to be the time points of complete recovery from the organic overloads. These samples were divided into four groups: control (1-no_shock_S1), group A (2-no_aug_S1 and 3-JJ_aug_S1), group B (4-aug_S1 and 5-sug_S3) and HPr-enriched cultures (7-NCT and 8-LJY) for metagenomic analysis (Figure 1C; Table 2).

**Table 2:** Sample information and recovery rates.

Group	Sample ID	Time point	Condition	Recovery rate (g COD/L/d)	HPr degradation rate (g Hpr/L/d)	HAc degradataion rate (g HAc/L/d)	CH_4_ production rate (ml CH_4_/d)
Control	1-no_shock_S1	S_1_ + 40d	No shock control	N/A	N/A	N/A	N/A
A	2-no_aug_S1	S_1_ + 40d	Shock only control	0.38^†^ (0.38–0.51)	0.36^†^ (0.28–0.41)	0.86^†^ (0.78–0.94)	8.42 (8.30–8.71)
	3-JJ_aug_S1	S_1_ + 40d	Bioaugmented with anaerobic sludge 0.94 g VS/L				
B	4-aug_S1	S_1_ + 40d	Bioaugmented with HPr-enriched culture 0.94 g VS/L	0.88^†^ (0.75–1.00)	0.77^†^ (0.59–0.83)	1.09^†^ (1.00–1.17)	10.41 (9.16–10.72)
	5-aug_S3	S_2_ + 30d					
HPr-enrichment	7-NCT	N/A	Enriched culture originally derived from ten full-scale AD*	N/A	N/A	N/A	N/A
	8-LJY	N/A	Enriched culture originally derived from twelve full-scale AD**	N/A	N/A	N/A	N/A

* Described in (Jannat et al., 2021).

** Unpublished data.

† Rates are significant between group A and B at a value of *p* < 0.01 from Kruskal-Wallis test.

Samples in group A had an organic shock but no bioaugmentation with Hpr-enriched cultures, while samples in group B had an organic shock and bioaugmentation with HPr-enriched cultures. Control sample did not have both organic shock and the bioaugmentation. Recovery, HPr degradation, HAc degradation, and CH_4_ production rates were obtained from (Rhee et al., 2021b) and represented as median with interquartile range (IQR).

In preparation for HPr-enrichment culture, two different systems from different sludge samples were selected due to their promising microbial community composition (Figure 1D; Table 2). One consortium (NCT) originated from ten different seeds (i.e., sludge from various full-scale anaerobic digesters) and was selected for the highest HPr oxidation and methane production rates among serial batch cultures (Kim et al., 2015). The cultures were fed with synthetic HPr wastewater ([S] = up to 3 g HPr/L) at 35°C. The top three cultures with a maximum HPr degradation rate of 0.08 g HPr/L/d were mixed to generate NCT (Jannat et al., 2021). The 8-LJY enrichment was collected from 250 ml semi-continuous laboratory anaerobic digesters treating synthetic HPr wastewater ([S] = 6.61 g HPr/L), operated at 37°C and 30 days of hydraulic retention time (HRT). The sample was collected during the steady-state period under the conditions described above.

### Sample collection, DNA extraction, and sequencing

Digestate samples were taken from the triplicate digesters and thoroughly mixed before DNA extraction. DNA was extracted from the digested samples as described previously (Rhee et al., 2021a, 2021b, 2021c). DNA libraries were prepared using the TruSeq Nano DNA kit (Illumina, San Diego, CA) and sequenced using a HiSeq4000 (Illumina; 2 × 150 bp paired-end run).

### Bioinformatics analysis

Metagenomic reads were quality-trimmed with SolexaQA, using phred quality cut-offs of 20 and 50 bp length after trimming (Cox et al., 2010). The reads were subsequently clipped to remove possible residual sequencing adaptor contamination (if any) using Scythe.^1^ The estimated abundance-weighted average coverage of each metagenomic dataset was calculated by Nonpareil version 3.20 with default parameters (Rodriguez-r and Konstantinidis, 2013). Mash, a tool that uses the MinHash dimensionality reduction method to compare overall sample-to-sample kmer composition (i.e., β-diversity; Ondov et al., 2016) was used to calculate pairwise distances between whole metagenomic datasets with a kmer = 25 option. β-diversity (i.e., taxa shared between samples) was calculated based on Mash distances and visualized using a non-metric multidimensional scaling (NMDS) plot with the vegan package in R v3.5.1 (Oksanen et al., 2015). Permutational multivariate analysis of variance (PERMANOVA) was exploited to reveal the effects of measured variables on the Mash distances (using the Adonis function of the vegan package; Oksanen et al., 2015). Trimmed paired-end reads were used for assembly using IDBA-UD (Peng et al., 2012; default options). The resulting contigs were binned into metagenome-assembled genomes (MAGs) using MaxBin and their completeness and contamination were assessed with CheckM (Wu et al., 2014; Parks et al., 2015). The quality of the MAGs was calculated as “Quality = Completeness – 5 × Contamination,” and MAGs with a quality score above 50 were selected for further analysis (Kim et al., 2022). GTDBtk was utilized to determine the most likely taxonomic classification and novelty rank of our good-quality MAGs (GTDB R202). dRep was used to estimate the genome-aggregate average nucleotide identity (ANI) among good-quality MAGs and an ANI 95% cutoff was used to generate ANI-based genomospecies (Olm et al., 2017). The relative abundance of MAGs was calculated by competitive read mapping (i.e., we concatenated all MAG sequences together in one file and competitively searched reads against this file) from bowtie2 alignments (Langmead and Salzberg, 2012). The relative abundance of MAGs among the metagenomes was normalized for our metagenomic dataset size and the average genome size of the microbial community samples using MicrobeCensus and expressed as genome equivalents (GEs; Nayfach and Pollard, 2015; Kim et al., 2022). Additionally, for a more conservative estimate, we calculated the 80% truncated coverage (TAD80) of MAG or gene abundance using the BedGraph.tad.rb script of the Enveomics collection (Rodriguez-R and Konstantinidis, 2016) to remove outlier genomic regions, in terms of sequence coverage, such as the rRNA and other multi-copy or recently horizontally transferred genes. Prodigal V2.6.3 (Hyatt et al., 2010) was used to predict genes from the MAGs, which were then annotated against the Trembl database (downloaded March 2022) and methylmalonyl CoA pathway genes from the HPr-oxidizing bacterium *Syntrophobacter fumaroxidans* using blastp (options of max_target_seqs 5). Matches to the references were filtered for best matching, using 40% identity and 70% sequence coverage over the alignment length as threshold values. To pinpoint the phylogenetic placement of ANIsp_017_JABUEY01 sp013314815 and the genomes of known syntrophic propionate degraders in methanogenic environment, we performed phylogenetic analyses for them. The genome sequences of known syntrophic propionate oxidizing bacteria were collected based on the list in the recently published review article (Westerholm et al., 2022). The 120 single-copy genes were identified, aligned, and concatenated using GTDB-tk with the “classify_wf” command. The maximum-likelihood phylogenetic tree was inferred based on this alignment using RAxML-NG v1.0.3 (Kozlov et al., 2019) with the best-fit model (i.e., LG + F + G4) selected by IQ-TREE v2.1.4 (Minh et al., 2020) and 1,000 bootstrap replicates. The tree was visualized with iTOL (Letunic and Bork, 2021).

## Results and discussion

### Characteristics of samples and resulting metagenomes

In our previous study, anaerobic digesters were operated under mesophilic conditions in semi-continuous mode. All digesters were overloaded, except for the no shock control bioreactors with two pulse-loadings at 10 g COD/L (S_1_) and 5 g COD/L (S_2_), respectively, for two different time points, using glucose (Figure 1; Table 2; Rhee et al., 2021b). We selected five different samples from anaerobic digesters for this metagenome studies together with two HPr-enrichment cultures (i.e., 7-NCT and 8-LJY) to assess the effect of our bioaugmentaion strategies on the shift of microbial community at the genomospecies level (See Table 2 and methods section for detailed information).

**Figure 2:**
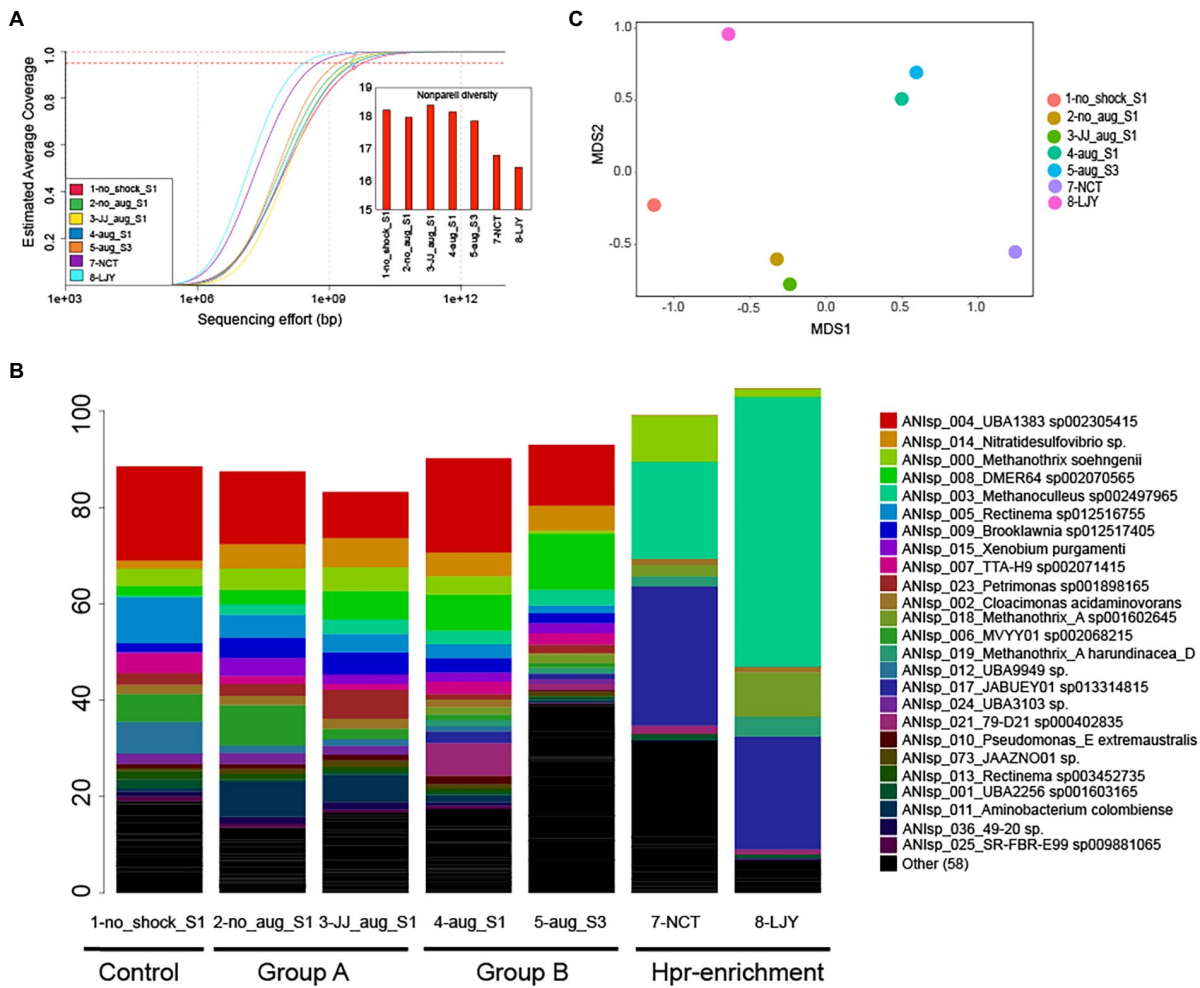
Microbial community diversity patterns and the relative abundance of genomospecies (MAGs). **(A)** Nonpareil curve and diversity of the samples used in this study (*n* = 7). The circles represent the estimated coverage of the microbial community sampled by the sequencing depth/effort applied. The projected line to the right of the circle represents the expected coverage for higher sequencing efforts. Dashed lines represent the sequencing needed for 95 and 99% coverage levels. Curves positioned more on the right represent more sequence-diverse metagenomes compared to curves positioned on the left, and this information is represented as Nonpareil diversity (see the embedded bar chart). **(B)** Relative abundance patterns of genomospecies (MAGs). The relative abundance was estimated based on the normalized TAD80 metric as described in the Materials and Methods section. MAGs were named with unique identifiers (e.g., ANIsp_numbers) followed by the closest relative of the MAG according to the GTDBtk results. For instance, ANIsp_055_Syntrophobacteraceae sp. was used for a MAG that represented a novel species of the *Syntrophobacteraceae* family based on the lowest rank (family in this case) shared with its best match against GTDB. **(C)** A non-metric multidimensional scaling (NMDS) plot of microbial community similarity based on MASH distances of whole metagenomes.

We obtained 47–54 million paired-ended reads after trimming per dataset (average read length ranged between 130 and 132 bp). Notably, the estimated abundance-weighted average coverage calculated by Nonpareil, an algorithm that examines the extent of overlapping reads within a dataset to determine the coverage and diversity, ranged between 89.6–96% (Figure 2A), suggesting adequate coverage for genome binning (Rodriguez-r and Konstantinidis, 2014). Assembly and population genome binning yielded a total of 200 high-quality (meaning: completeness – 5 × contamination ≥50) metagenome-assembled genomes (MAGs), representing 83 distinct genomospecies at 95% genome-aggregate average nucleotide identity (ANI). Taxonomic classification using GTDBtk suggested that 35/83 genomospecies represent novel species or higher taxonomic ranks (75/83 novel species of higher taxonomic ranks per NCBI classification; Supplementary Table S1). Specifically, 26 were predicted to be novel species of a previously described genus, 7 were novel species of a previously described family, 1 was novel species of a previously described order and 1 was novel species of a previously described class. MAGs were named with unique identifiers (e.g., ANIsp_numbers) followed by the closest relative of the MAG according to the GTDBtk results. For instance, ANIsp_055_Syntrophobacteraceae sp. was used for a MAG that represented a novel species of the *Syntrophobacteraceae* family based on the lowest rank (family in this case) shared with its best match against GTDB. Notably, all 200 MAGs together recruited an average of 92.2 ± 7.3% of the total reads in each metagenome (Figure 2B), revealing that the MAGs represented most of the microbial communities sampled, consistent with the relatively high coverage values obtained *via* Nonpareil.

### Shifts in microbial community structure in bioaugmentation-treated anaerobic digesters

Whole-metagenome comparisons based on MASH distances (i.e., overall kmer similarity) suggested a clear separation of microbial communities in group A (i.e., with bioaugmentation of conventional anaerobic sludge and shock only control) from those of group B (i.e., with bioaugmentation of HPr-enriched cultures; Table 2; Figure 2C). Interestingly, bioaugmentation of conventional anaerobic sludge (i.e., 3-JJ_aug_S1) and shock only control sample (i.e., 2-no_aug_S1) not only showed almost the same recovery rate (average of 0.4416 ± 0.0987 vs. 0.4407 ± 0.1636 g COD/L/d) but also very similar microbial community structure (Figure 2C), while the microbial communities of two samples in group B (i.e., with bioaugmentation of HPr-enriched cultures) clustered together (Figure 2C). This suggested the different treatment regimes selected for the presence of unique taxa in each group and the bioaugmentation of HPr-enriched cultures significantly affect the shift of microbial community structure and the performance of anaerobic digesters after the organic loading shock. Permutational multivariate analysis of variance (PERMANOVA), a non-parametric test to evaluate the effects of variables through partitioning of variance, was performed to investigate the effect of four different conditions of samples such as non-organic-shock control, bioaugmentation with conventional anaerobic sludge, bioaugmentation of HPr-enriched consortia, and HPr-enriched consortia (Table 2). About 88% of the microbial community variation observed among samples was explained by the conditions (*R*^2^ value of 0.87657 for conditions and a value of *p* of <0.01, PERMANOVA). It should be noted that our sample size for each condition is small and unbalanced. Thus, the differences observed between conditions need to be further corroborated with more samples in the future.

### Identifying key syntrophic propionate-oxidizing bacteria and methane-producing archaea for superior recovery responses to organic overload

The HPr degradation efficiency and rate were 99.5% and 0.219 g propionic acid/L/d, respectively (Unpublished data). This laboratory anaerobic digester had previously been inoculated with a mixture of samples collected from twelve full-scale anaerobic digesters treating food wastewater and/or sewage sludge (Lee et al., 2021). Strikingly, we observed that these two distinct HPr-enrichment consortia selected for the same two microorganisms (i.e., ANIsp_003_Methanoculleus sp002497965 and ANIsp_017_JABUEY01 sp013314815), which represented a substantial proportion of the microbial communities of both enrichment systems, at about 49 and 79% abundance in sludge samples 7-NCT and 8-LJY, respectively (Figure 2B; Supplementary Table S2). JABUEY01 sp013314815 belongs to the *Syntrophobacteraceae* family and its genome sequence was recently recovered from metagenomes of artesian water collected at a 2.8 km deep oil exploration borehole (5P) in Western Siberia, Russia (i.e., Ch74 genome; Kadnikov et al., 2020). While *Syntrophobacteraceae* species are known to be acetate and propionate-degrading sulfate reducers in natural environments such as paddy soil (Kadnikov et al., 2020), it has yet to be reported that JABUEY01 sp013314815 can oxidize HPr at high rates in AD processes.

**Figure 3:**
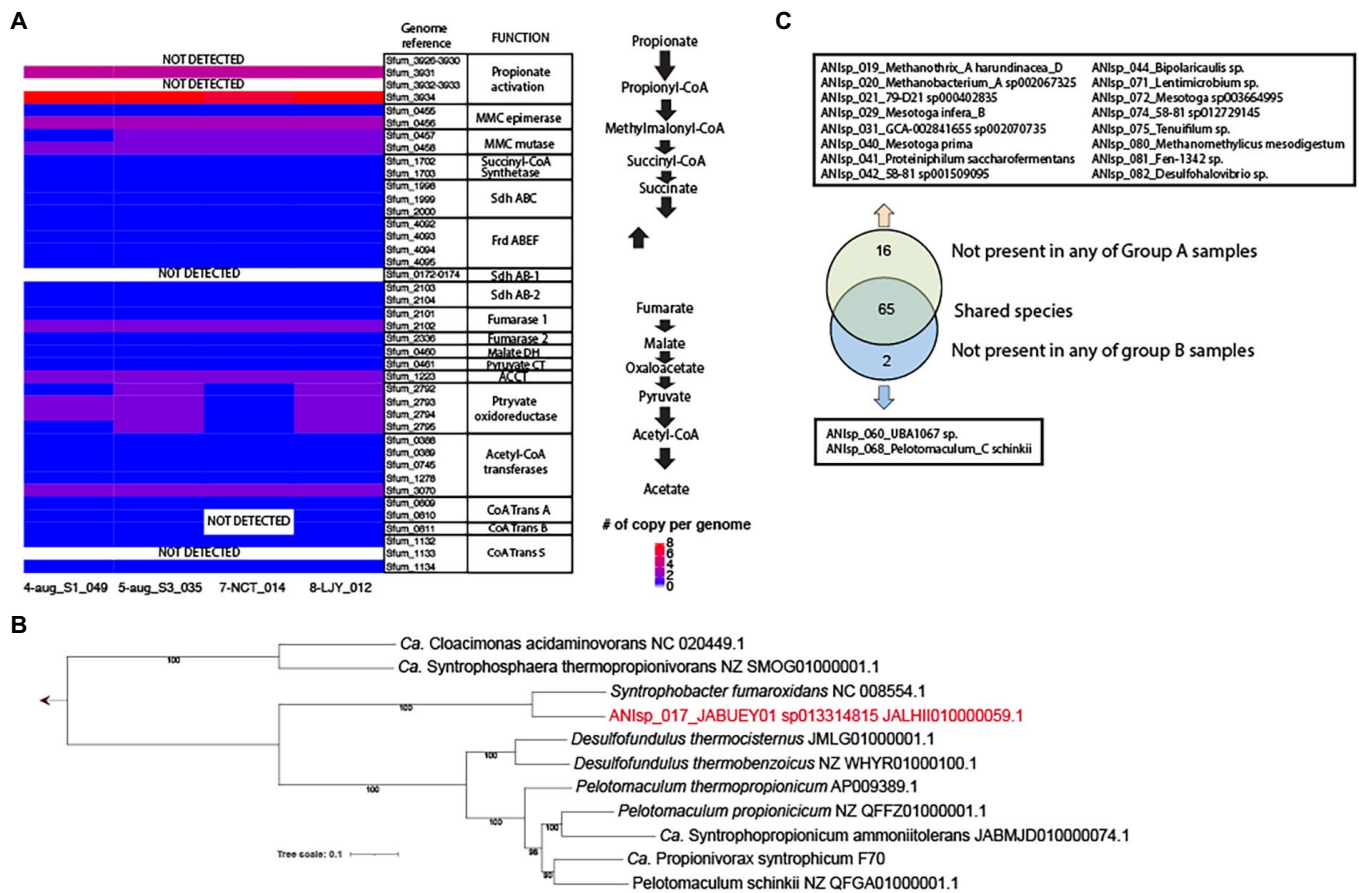
Presence and absence of MAGs in group A vs. group B metagenomes and potential for propionate degradation in key species. **(A)** A copy number of genes per genome for the methylmalonyl-CoA pathway in 4 MAGs of ANIsp_017_JABUEY01 sp013314815. MMC, methylmalonyl-CoA; Sdh, succinate dehydrogenase; Frd, fumarate reductase; DH, dehydrogenase; CT, carboxyltransferase; ACCT, acetyl-CoA carboxyltransferase; CoA Trans, coenzyme A transferase. **(B)** Phylogenetic genome tree of ANIsp_017_JABUEY01, which is highlighted in red, and known propionate degraders. The genome sequence of *Ca.* Propionivorax syntrophicum F70 was kindly provided by Dr. Morten Simonsen Dueholm. **(C)** A diagram representing presence and absence of MAGs in group A vs. group B metagenomes.

Our genomic analysis of all 4 MAGs of ANIsp_017_JABUEY01 sp013314815 (i.e., 4-aug_S1_049, 5-aug_S3_035, 7-NCT_014, and 8-LJY_012) that were recovered from both group B samples (See Supplementary Table S1 for the full list of MAGs) and the two HPr-enrichment systems found nearly complete sets of the methylmalonyl-CoA pathway for HPr oxidation. These proteins all exhibited strong homology (> 40% amino acids identity and > 70% sequencing coverage) to those found in the well-characterized propionate oxidation pathway of *Syntrophobacter fumaroxidans* (Figure 3A; Sedano-Núñez et al., 2018). The only components of the pathway that were lacking were several genes predicted to be involved in propionate activation, such as the propionate CoA-transferase (*pct*). Nevertheless, at least partial sets of three CoA transferases were found in these MAGs, and a previous study detected several CoA transferases besides Pct in proteomics data obtained from a co-culture of *S. fumaroxidans* in association with several different methanogens (i.e., *Methanobacterium formicicum* and *Methanospirillum hungatei*) grown with 30 mM of propionate (Sedano-Núñez et al., 2018). Thus, we contend that other potential genes for propionate CoA-transferase activity besides *pct* can mediate propionate activation *in vivo*. On a final note, all four of these MAGs showed >99% average nucleotide identity (ANI) demonstrating that the same strain of JABUEY01 sp013314815 was present in both the bioaugmented (i.e., group B) and HPr-enrichment systems. Phylogenetic placement of single-copy genes alignmentof ANIsp_017_JABUEY01 sp013314815 and other known propionate degraders in methanogenic systems suggested that ANIsp_017_JABUEY01 sp013314815 clustered with *Syntrophobacter fumaroxidans* (Plugge et al., 2012) among the other known propionate degraders (Figure 3B).

*Methanoculleus* is a hydrogenotrophic methanogen and has been implicated in syntrophic HPr conversion in AD processes, particularly at inhibitory concentrations of HPr (Cao et al., 2021). This is consistent with findings from our research that ANIsp_003_*Methanoculleus* sp002497965 became dominant together with ANIsp_017_JABUEY01 sp013314815 in our two HPr enrichment systems. The presence of both these microorganisms in the two digestate samples that efficiently mineralized HPr implicates both species as the primary drivers of the rapid syntrophic HPr oxidation we observed. While the relative abundance of ANIsp_003_*Methanoculleus* sp002497965 remained similar in both group A and group B samples after organic loadings (i.e., 2–3% of the total microbial communities), the relative abundance of ANIsp_017_JABUEY01 sp013314815 in group B was 60× higher than in group A (i.e., the average relative abundance of 1.7% vs. 0.029%). Thus, maintaining a higher relative abundance of ANIsp_017_JABUEY01 sp013314815 in the AD systems enhanced the recovery rate in our organic overloaded AD processes. This effect was so pronounced that we observed double the rate of HPr removal and recovery from organic overload in the group B bioreactors that were supplemented with our putative syntrophic HPr oxidizers ANIsp_017_JABUEY01 sp013314815 and ANIsp_003_Methanoculleus (value of *p* <0.01, Kruskal-Wallis test; Table 2).

To identify additional taxa that might be responsible for the different recovery rates we observed between groups A and B, the relative abundance of all genomospecies was estimated using read recruitment analysis and the TAD80 metric as described in the Methods section above. Out of 83 genomospecies, 16 were not present in any of group A samples (i.e., No bioaugmentation with HPr-enriched cultures) but present in all group B samples (i.e., bioaugmentation with HPr-enriched cultures), and 2 were not present in any of group B samples but were present in all group A samples (Figure 3C; Supplementary Table S2). This result was consistent with the β-diversity calculated from MASH distances, providing strong support for the contention that the impressive resiliency of group B to organic overloading was conferred by the microbial communities they were supplemented with (i.e., the HPr-enrichment bioaugmented bioreactors; Supplementary Table S2; Figure 2B; Figure 3C). Out of the 16 genomospecies absent in the group A samples, three, including ANIsp_019_*Methanothrix*_A *harundinacea*_D (*Methanosaeta harundinacea* for NCBI classification), ANIsp_021_79-D21 sp000402835 (unclassified species of *Synergistetes* for NCBI classification), and ANIsp_040_*Mesotoga prima* (unclassified species of *Mesotoga* for NCBI classification), showed >1% of relative abundance after organic overloading in all group B samples (Figure 2B; Supplementary Table S2).

*Methanosaeta harundinacea* is an acetoclastic methanogen that can digest HAc to yield CH_4_ (Smith and Ingram-Smith, 2007). Given the high relative abundance of ANIsp_019_*Methanothrix*_A *harundinacea*_D in the two HPr-enrichment systems (i.e., 3.1 ± 1.4%) and its exclusive presence in group B AD samples at >1% relative abundance, this species might play an important role in the elevated HAc degradation rate observed in group B treatments compared to group A. The increased HAc degradation rate also plays a role in the enhanced recovery rate we observed after organic overloading (Table 2). ANIsp_021_79-D21 sp000402835 (unclassified species of *Synergistetes* for NCBI classification) showed a similar relative abundance pattern to that of ANIsp_019_*Methanothrix*_A *harundinacea*_D (i.e., higher relative abundance in both HPr-enrichment systems and exclusively present in group B digestate samples). The genome of this species was recovered from a methanogenic bioreactor degrading terephthalate (Nobu et al., 2015) but there have been no previous studies regarding its metabolic potential in AD processes yet. We attempted to search its genomic potential for both HPr and butyric acid oxidation by searching the predicted genes of ANIsp_021_79-D21 sp000402835 against Trembl database, *S. fumaroxidans* HPr oxidation genes (methylmalonyl-CoA pathway), and *Desulfoscipio gibsoniae* butyric acid oxidation genes but were able to find only a few genes in both pathways suggesting this organism performs other functions than HPr and butyric acid oxidation in the AD processes in response to organic overloading. It cannot be excluded, however, that genes from uncharacterized organisms mediated HPr and butyric acid oxidation pathways. *Mesotoga prima* was reported to efficiently ferment carbohydrates to acetic acid (Nesbø et al., 2012). While ANIsp_040_*Mesotoga prima* was absent in one HPr-enrichment (i.e., 7-LJY) and was only present in another HPr-enrichment (i.e., 8-NCT) with 0.24% of relative abundance, its relative abundance in all group B samples was higher than 1% and absent in all group A samples. This result suggested that this species may be associated with the fermentation of glucose or other carbohydrates in our AD processes. This hypothesis will require further testing in future experiments.

## Conclusion and outlook

Key microorganisms that are responsible for HPr oxidation were recovered as MAGs from organic overloaded anaerobic digesters and HPr-oxidizing enrichments. Our results suggested at least three vital players (a putative syntrophic HPr-oxidizing *Syntrophobacteraceae* and hydrogenotrophic/acetoclastic methanogens) that have contributed to the enhanced HPr oxidation, and therefore rapid recovery, rates observed in the bioaugmented systems. We propose that these novel organisms would make for an effective microbial cocktail that can be added during the start-up, organic-overload, and unstable operation phases of AD processes. Furthermore, we found that all of our AD systems together with two HPr-enrichments contain ANIsp_002_Cloacimonas_acidaminovorans (*Candidatus* Cloacimonas acidaminovorans), which are known to be syntrophic HPr oxidizing bacteria (Nobu et al., 2015; Figure 3B), with higher than 1% of relative abundance except for 5-aug_S3 (0.35%) together with other hydrogenotrophic methanogens such as *M. soehngenii*. This observation also suggested that there might be multiple different syntrophic consortia for HPr oxidation in our AD systems. Another future experiments for bioaugmentation using these HPr oxidizing bacteria (e.g., *Ca.* Cloacimonas acidaminovorans, *S. fumaroxidans*, *Pelotomaculum* spp.) and methanogens (e.g., *M. soehngenii*) would be required to find the best consortia for various AD systems. Lastly, our results and datasets in this manuscript would serve as a community resource because these contain a total of 200 MAGs representing 83 genomospecies from AD systems and 35 of them represent previously unknown species.

## Limitations

Our study has limitations. Most notably, our practice of pooling triplicate samples together before DNA extraction can mask the variation in biological replicates. In other words, it is unclear if our key HPr oxidizing players were present in higher relative abundance after the recovery from organic overload in all of our group B replicates (i.e., Bioaugmented with HPr-enriched cultures) or in only one of these replicates. Even if it is the latter case, there is a great potential for the key players to be an effective microbial cocktail because we observed not only higher abundance of the key players (e.g., ANIsp_017_JABUEY01 sp013314815) after the recovery from two different organic overloadings (i.e., group B samples) compared to control and group A samples, but also their very high relative abundance in two different HPr enrichment cultures. Further, we only have a limited number of samples to conduct proper statistical analyses (e.g., correlation analysis between biological factors and abiotic factors). Future research with more samples is needed to reveal the specific roles of the key players and their interaction with native microbial community members in recovery from organic overloaded ADs.

## Data availability statement

All metagenome sequences and biosamples for MAGs used in this study are available in NCBI, under BioProject number https://dataview.ncbi.nlm.nih.gov/object/PRJNA818325?reviewer=ct8o10fce7o8atd403pdgio1hg PRJNA818325. The MAG sequences recovered in this study are available under GenBank accession numbers JALHCI000000000-JALHJZ000000000.

## Author contributions

MK: investigation, methodology, formal analysis, and writing – original draft. CR and JS: investigation, and writing – review and editing. MW: writing – review and editing. JL: resources, and writing – review and editing. SS: conceptualization, supervision, funding acquisition, and writing – review and editing. All authors contributed to the article and approved the submitted version.

## Funding

This work was supported by the National Research Foundation of Korea (NRF) grant funded by the Korea government (Ministry of Science, ICT and Future Planning; No. 2017R1C1B1011494, 2020R1C1C1013643, and 2022R1C1C2007902). This work was also supported by Korea Environment Industry & Technology Institute (KEITI) through its Ecological Imitation-based Environmental Pollution Management Technology funded by Korea Ministry of Environment (MOE; 2019002790004).

## Conflict of interest

The authors declare that the research was conducted in the absence of any commercial or financial relationships that could be construed as a potential conflict of interest.

## Publisher’s note

All claims expressed in this article are solely those of the authors and do not necessarily represent those of their affiliated organizations, or those of the publisher, the editors and the reviewers. Any product that may be evaluated in this article, or claim that may be made by its manufacturer, is not guaranteed or endorsed by the publisher.

## Footnotes

^1^ https://github.com/vsbuffalo/scythe
